# Anticipated barriers and facilitators for implementing smart inhalers in asthma medication adherence management

**DOI:** 10.1038/s41533-023-00343-w

**Published:** 2023-05-20

**Authors:** Susanne J. van de Hei, Nilouq Stoker, Bertine M. J. Flokstra-de Blok, Charlotte C. Poot, Eline Meijer, Maarten J. Postma, Niels H. Chavannes, Janwillem W. H. Kocks, Job F. M. van Boven

**Affiliations:** 1 Department of Health Sciences, University Medical Center Groningen, University of Groningen, Groningen, The Netherlands; 2 Groningen Research Institute for Asthma and COPD (GRIAC), Groningen, The Netherlands; 3 General Practitioners Research Institute, Groningen, the Netherlands; 4 Department of Pediatric Pulmonology and Pediatric Allergology, Beatrix Children’s Hospital, University of Groningen, University Medical Center Groningen, Groningen, the Netherlands; 5 Department of Primary Care and Public Health, Leiden University Medical Center, Leiden, The Netherlands; 6 National eHealth Living Lab, Leiden, The Netherlands; 7 Observational and Pragmatic Research Institute, Singapore, Singapore; 8 Medication Adherence Expertise Center of the northern Netherlands (MAECON), Groningen, The Netherlands; 9 Department of Clinical Pharmacy & Pharmacology, University Medical Center Groningen, University of Groningen, Groningen, The Netherlands; 10 Centre for Medicine Use and Safety, Monash Institute of Pharmaceutical Sciences, Monash University, Melbourne, VIC Australia

**Keywords:** Health policy, Asthma

## Abstract

Smart inhalers are electronic monitoring devices which are promising in increasing medication adherence and maintaining asthma control. A multi-stakeholder capacity and needs assessment is recommended prior to implementation in healthcare systems. This study aimed to explore perceptions of stakeholders and to identify anticipated facilitators and barriers associated with the implementation of smart digital inhalers in the Dutch healthcare system. Data were collected through focus group discussions with female patients with asthma (*n* = 9) and healthcare professionals (*n* = 7) and through individual semi-structured interviews with policy makers (*n* = 4) and smart inhaler developers (*n* = 4). Data were analysed using the Framework method. Five themes were identified: (i) perceived benefits, (ii) usability, (iii) feasibility, (iv) payment and reimbursement, and (v) data safety and ownership. In total, 14 barriers and 32 facilitators were found among all stakeholders. The results of this study could contribute to the design of a tailored implementation strategy for smart inhalers in daily practice.

## Introduction

While effective inhaler medication is available, nearly half of all patients with asthma do not have their condition under control^1–3^. Suboptimal control is associated with increased symptoms, health utilisation and economic burden^4–7^. One of the main reasons of inadequate asthma control is poor adherence to controller inhalers^8,9^. Many factors contribute to poor medication adherence, such as forgetfulness (erratic non-adherence), difficulties understanding the instructions—such as inhaler technique—or the specifics of the regimen (unwitting non-adherence), or unhelpful perceptions of medication and illness (intelligent non-adherence)^7,10^.

Different methods exist to determine non-adherence in patients with asthma (e.g. checking dispensing records, using questionnaires, canister weighing or simply asking patients if they are adherent to the prescribed medication), but it is known that those methods often overestimate true adherence rates^11^. A more objective method to determine non-adherence is through electronic monitoring devices (EMDs), which use electronic sensors attached to, or integrated in, inhalers to detect inhaler use^12^. Various EMDs have been developed, ranging from simple devices that only track medication use to more advanced devices, also known as “smart inhalers”^11^. Smart inhalers offer varying additional functionalities via smartphone applications, such as sending reminders and/or motivational messages, providing real-time personalised feedback (e.g. on actual usage or inhaler technique), or monitoring asthma symptoms.

Smart inhalers could help healthcare professionals (HCPs) in providing guided self-management (i.e. providing patients with skills to empower them to effectively manage their chronic disease on a day-to-day basis)^7,13^. Several studies have found that electronic inhaler reminders significantly increase medication adherence compared to standard care^14–20^. However, an improvement in asthma control has only been found in children with asthma^16^.

Despite the accumulating evidence for the effectiveness and value of eHealth-based self-management applications for asthma, many have not been implemented successfully in daily practice^21,22^. Failure of adoption has several reasons, such as data privacy issues or the lack of skills or motivation^23–25^. A Delphi survey among HCPs and policy makers found that pros to electronic monitoring of inhaler use in asthma included evidence to aid discussions between clinician and patient and an increase of patient involvement and motivation. Cons rated as most important were the lack of evidence on effectiveness, and the uncertainty on who is responsible for data handling^26^. Another study found that barriers to implementation of electronic medication monitoring in paediatric asthma care perceived by HCPs are increased workload and technology syncing issues^27^. More generally, the experiences of end-users and implementers of mHealth technologies for management of chronic noncommunicable diseases in young adults has been evaluated in a systematic review, which found that perceived barriers to implementation of mHealth technologies included reimbursement and funding, integration into the current work flow, readiness for change and data security^28^. Yet, little is known about perceived facilitators and barriers from the perspective of patients with asthma and developers of smart inhalers regarding the implementation of smart inhalers.

Of note, it is recommended to perform a multi-stakeholder capacity and needs assessment prior to implementation of health innovations in a real-world setting, in order to identify potential barriers and facilitators to implementation from the perspective of all stakeholders^29,30^. This assessment can help in designing an implementation strategy, a “method or technique used to enhance the adoption, implementation, and sustainability of a clinical programme or practice”^31^. Therefore, the aim of the current study was to explore different stakeholder perceptions and to identify any anticipated facilitators and barriers associated with the implementation of smart inhalers in the Dutch healthcare system.

## Methods

### Study design

We explored the perspectives of relevant stakeholders on the implementation of smart inhalers for asthma in daily practice in the Netherlands and its anticipated barriers and facilitators, using a qualitative design^32^. Data were collected through semi-structured interviews and focus group discussions which took place online using Zoom video conferencing software in the period from April 2020 to June 2020 in the Netherlands. The medical ethics committee of the University Medical Center Groningen (UMCG) deemed that formal medical ethical approval was not required, as this study did not fall under the Dutch Medical Research Involving Human Subjects Act (METc number 2020/145). This study is reported in accordance with the “Consolidated criteria for reporting qualitative studies” (COREQ) statement^33^. The study was registered in the Netherlands Trial Register with no. NL8495 prior to data collection.

### Participants

Four groups of participants, representing different key stakeholders involved in the implementation of smart inhalers, were defined: (i) patients with asthma, (ii) HCPs, (iii) policy makers (i.e. representatives from public health institutions/decision makers, health insurance companies and patient organisations), and (iv) smart inhaler developers (i.e. representatives from pharmaceutical companies and medical device companies involved in the development and manufacturing of smart inhalers). Selection of policy maker and developer representatives was based on their function within the company or institution (i.e. most knowledgeable on the topic of interest)^34^. Purposive sampling was used to select HCPs for the focus group discussion (e.g. occupation). Convenient sampling was used to select patients with asthma (i.e. all eligible patients with asthma that were interested could participate). Inclusion criteria were age 18 years and older, self-reported asthma, the use of controller inhalation medication for asthma and adequate oral fluency in Dutch.

### Procedure

#### Focus group discussions

To explore patients’ and HCPs’ opinions, two separate focus group discussions were organised. We used focus group discussions instead of individual interviews, as a focus group discussion allows for interaction among participants which provides insight in (lack of) consensus between participants on a certain topic. Two focus group discussion guides were developed, based on the literature^26–28,35^, and our own experiences studying the use of smart inhalers (Supplementary Methods 1). The focus group discussions were held using Zoom and led by N.S. (moderator, “science business and policy” master student) with S.J.v.d.H. (general practitioner in training and PhD-student) and B.M.J.F.-d.B. (PhD) assisting in data collection (observers). The moderator and observers had no involvement in patient care of the participating patients. The participants of both focus group discussions had no personal background information on the interviewers (other than a brief introduction during the focus group discussions), except for one HCP who was a known contact of one of the observers. The focus group discussions were conducted in Dutch and lasted 86 min (patients) and 85 min (HCPs), including a 10-min break. Field notes were made during the focus group discussions. Focus group discussions were recorded and transcribed verbatim. Participants of the patient focus group discussion received a €25 gift coupon as compensation for their time spent, HCPs that participated were offered €100 to compensate for their time investment. Using an extensive single focus group session for patients and for HCPs, it was anticipated that a sufficient level of data saturation would be reached. However, data saturation may have been increased by organising the focus groups with even more patients and HCPs.

#### Interviews

The opinions of policy makers and developers of smart inhaler were collected by individual semi-structured interviews (Supplementary Methods 2). We opted for interviews so that participants felt free to speak openly (i.e. no presence of business competitors), and for logistical reasons (i.e. busy schedules). N.S. conducted all interviews using Zoom, except for one telephonic interview with a participant that worked in a company that did not support video calling. One interview was held in English, the rest of the interviews were conducted in Dutch. Interviews lasted 50 min on average (range 34–77 min). Interviews were recorded and transcribed verbatim.

#### Recruitment

Patients with asthma were recruited through social media advertisement. Upon expression of interest, a patient information sheet was sent by email. The sheet contained information about the concept “smart inhaler”, the purpose of the study and inclusion criteria, it explained that participation is voluntary and can be ended at any time, that the focus group discussion would be audio-recorded and that data will be used confidentially and anonymously. Written informed consent was obtained from participating patients prior to the focus group discussion. Furthermore, all patient participants completed a questionnaire prior to the focus group discussion assessing age, sex, education, age of onset of respiratory symptoms, year of asthma diagnosis, and respiratory medication use including years of use and the Asthma Control Questionnaire (ACQ-6). The ACQ consists of six items, each rated on a 7-point scale (0–6 points, with 6 indicating poor control); five questions concern symptoms and one question concerns the frequency of short-acting β2-agonist use^36^. Patients with a score below 0.75 were considered as having controlled asthma and those with a score equal to or greater than 1.5 points were considered as having uncontrolled asthma^37^.

HCPs working in professions that may be involved with smart inhalers in the future were invited to participate in the focus group discussion via e-mail through the authors’ network (e.g. paediatricians, pulmonologists, general practitioners, pharmacists and nurses working in the respiratory field), as were relevant policy makers in the Netherlands and smart inhaler developers. The e-mail contained information on smart inhalers, the purpose of the study and voluntary participation. At the start of the focus group discussion or interview, it was explained that the focus group discussion would be audio-recorded and that data will be used confidentially and anonymously. All participants provided verbal or written informed consent.

### Data analysis

Data were analysed according to the Framework method, using an inductive approach^32,38^. First, two persons (N.S. and S.J.v.d.H.) read the transcripts several times independently to familiarise themselves with the data. All transcripts were independently coded by N.S. and S.J.v.d.H. using Dedoose software (version 9.0). After coding three randomly selected transcripts, the initial analysis and coding tree were discussed and adapted. The remaining transcripts were then coded using the coding tree. Both authors independently adapted the coding tree when data did not fit in an existing code. After all transcripts were coded, the coding tree was discussed again and finalised (see Supplementary Methods 3). Subsequently, any discrepancies in coding were discussed between the authors until consensus was reached. Due to different backgrounds of the coding authors, different viewpoints were ensured, which limited the risk of confirmation bias. After coding, data for each participant group were charted into tables based on the codes and interpreted to identify themes in the data related to the research question by S.J.v.d.H. and N.S. After reviewing, discussing and refining the themes, the transcripts were re-read by S.J.v.d.H., to re-examine the themes (i.e. to ensure that the themes reflect the entire body of data and no relevant aspects have been missed).

### Reporting summary

Further information on research design is available in the Nature Research Reporting Summary linked to this article.

## Results

### Study population

In total, 20 patients with asthma were interested in participation, 13 met inclusion criteria and nine participated in the focus group discussion (four did not reply to our follow-up e-mails). Invitations were sent to 20 HCPs to which 17 responded and seven were able to participate in the focus group discussion. Furthermore, seven Dutch policy makers (working for different organisations, including the Dutch Ministry of Health Welfare and Sports, Dutch Healthcare Institute (ZIN), the Lung Foundation Netherlands and a large Dutch health insurer) were invited and four participated. Finally, four smart inhaler developers working for four different international companies with headquarters across three different countries (United Kingdom, Italy and Switzerland) were invited and all participated. In total, 24 people participated in the study: 8 in the individual interviews and 16 in the focus group discussions. A diverse group of patients was included in terms of age, ACQ scores and education level, but no male patients participated (Table 1). At least one HCP was included from all relevant professions (Table 2).

**Table 1:** Characteristics of patient participants.

Patients with asthma (*n* = 9)					
Patient	Age (years)	Sex	Education	Age of onset of respiratory symptoms	ACQ-5 score
1	52	Female	Vocational degree	12	2.0
2	26	Female	Postgraduate degree	14	1.0
3	26	Female	Postgraduate degree	2	0.2
4	41	Female	Vocational degree	30	0.6
5	28	Female	Postgraduate degree	16	1.6
6	25	Female	Bachelor degree	5	2.0
7	30	Female	Bachelor degree	5	1.2
8	24	Female	Bachelor degree	10	1.8
9	60	Female	Secondary school	45	1.2
Mean (SD)	34.7 (13.3)	–	–	15.4 (13.8)	1.3 (0.6)

*SD* standard deviation.

**Table 2:** Characteristics of healthcare professional participants.

Healthcare professionals (*n* = 7)		
HCP	Sex	Profession
1	Male	General practitioner
2	Female	Nurse working in respiratory field
3	Female	Nurse working in respiratory field
4	Male	Paediatrician
5	Female	Pharmacist
6	Male	Pulmonologist
7	Female	Pulmonologist

*HCP* healthcare professional.

### Identified themes

Five themes related to anticipated barriers and facilitators for successful smart inhaler implementation were identified from the data: (i) perceived benefits, (ii) usability, (iii) feasibility, (iv) payment and reimbursement, and (v) data ownership. Each theme will be further specified in the next section and all identified barriers and facilitators are summarised in Table 3. Supporting quotes (Q1–Q25) can be found in Table 4.

**Table 3:** Identified barriers and facilitators summarised by theme.

**Theme 1: Perceived benefits**	
F	Availability of functionalities (PT, HCP, D)
F	Patients being motivated to use smart inhalers (HCP)
B	Regular confrontation with disease for patients (HCP)
B	Alternative options to improve asthma care available (PM)
B	Patient characteristics: well-controlled asthma (PT, D), lack of need for change (PT), higher age (PT, PM)
F	Use of smart inhalers supported by HCP (PT)
F	Evidence on improved clinical outcomes (HCP)
F	Smart inhalers implemented in guidelines (HCP)
F	Evidence of benefits for individual health, population health and cost reduction (PM)
F	Evidence on which subgroup(s) would benefit (PM)
F	Start-stop criteria (PM)
F	Evidence on feasibility of implementation (PM)
**Theme 2: Usability**	
***2.1. User-friendliness***	
F	Inhaling is as easy as without smart inhaler (PT, HCP)
F	Limited frequency of push messages, no pedantic character (PT, HCP)
F	Intuitive installation and use, not requiring much effort (PT, HCP, D)
F	Easy to prescribe (HCP)
F	Passive data collection, little effort for patients and HCPs (PT, HCP, D)
F	Possibility to personalise the app (PT, HCP, D)
F	Involvement of all stakeholders in development process (D)
***2.2. Compatibility***	
B	Lack of compatibility on different levels (PT, HCP, PM, D)
***2.3. Education and support***	
F	Ability to choose the form of support (when setting up and using smart inhalers) (HCP)
F	Possibility to contact HCP in case of problems with smart inhaler (PT)
F	Clear protocol and one responsible person within organisation in case of problems (HCP)
F	Trained team at HCP level (HCP, D)
F	Helpdesk, troubleshooting material and intuitive instructions in app (D)
**Theme 3: Feasibility**	
F	Minimal time investment and fit in daily routines (PT)
B	Limited consultation time (HCP)
F	Small target group (HCP)
B	Lack of environmental sustainability of smart inhalers (HCP, PM)
F	Agenda setting (HCP, PM, D)
F	(National) platform for smart inhalers provided by policy makers (D)
B	(Dutch) legislation regarding promotion of medication/medical devices (HCP, D)
F	Healthcare organisations need to be willing to change workflows (PM)
B	Development of smart inhalers evolves more slowly than expected (D)
**Theme 4: Payment and reimbursement**	
B	Lack of reimbursement (PT, HCP)
F	Clearly defined target group to minimise costs (PM)
B	Current financial incentive in (Dutch) health care system(s) (i.e. production could decline due to innovation) (PM)
B	Difficult to decide which technologies should be reimbursed due to rapid evolvement of technologies (PM)
F	Reimbursement of invested time for HCPs (D)
**Theme 5: Data safety and ownership**	
F	Knowledge on who has access to data (PT)
F	Proper data security (PT)
B	Commercial interest (PT, PM)
B	Uncertainty on who has access to data and on what developers will use data for (PM)
F	Providing clear information to patients on data storage (where, purpose, who has access) (D)
F	Data sharing (PT, HCP)
B	Data sharing (PT, HCP)

*B* barrier, *D* developer, *EHR* electronic health records, *F* facilitator, *HCP* healthcare professional, *PM* policy maker, *PT* patient.

**Table 4:** Quotes.

	Quote	Participant group
**Theme 1: Perceived benefits**		
1	*“We must be very realistic that some of the patients do not want to be patients, and that they don’t want to be constantly confronted with their disease. And that they don’t like it when they receive emails.”*	HCP
2	*“But then I would like it to be researched, that it really works well, and that pulmonologists support it, and not when you come up with symptoms, that they say: “Yeah, that app doesn’t work well anyway, so we don’t do anything with it”.”*	Patient
3	*“And then of course the question arises: what exactly is the cost-effectiveness and what is the added value? And the second question is: do you need such a device permanently or it is temporary? Because that is also important to me.”*	Policy maker
**Theme 2: Usability**		
***2.1. User-friendliness***		
4	*“[A reason not to use it is] if it is a very complicated app and I have to put in a lot of effort to complete everything. It really must be a tool that is easy to use. Because otherwise, I know that I might use it fanatically for a few days, and then I’ll quit. So that’s really a requirement for me.”*	Patient
5	*“For example, if you are on a drug that you don’t have to take every day, you shouldn’t get a reminder every day. If that [taking medication every day] is very important, then yes. And then it would be good if certain messages are sent, but I think it should be adapted to what you want to achieve for that patient.”*	Developer
6	*“We’re working with pharmacies, we’re working with hospitals, we’re working with GPs, we’re working with insurance companies. I mean, the delivery and implementation of the system, it has to include all of them and, you know. All of these different stakeholders are part of the feedback loop basically, eventually, and of the product development process.”*	Developer
***2.2. Compatibility***		
7	*“There are a lot of different inhalers, and if there are actually a lot of different smart inhalers with all kinds of functionalities and different apps, well, I think it will quickly get tiresome to get started. There has to be some sort of uniformity.”*	HCP
8	*“I think if you want to implement it, you need to have an overview of which inhalers are most prescribed. And that you develop a smart inhaler for those [inhalers]. Because there are many types of inhalers, and I think it’s better to have a few good smart inhalers, then to make one for all the dozens of types of inhalers out there.”*	HCP
***2.3. Education and support***		
9	*“Well, I think its easiest to contact the pulmonary nurse [in the general practice] for that [in case of problems with smart inhalers]. Or the general practitioner. I think it is cumbersome to contact the manufacturer. And that it is less easy to connect with them [the manufacturers]. So, I wouldn’t really need that.”*	Patient
10	*“I think training for the whole team [is needed]. I think it would be good, if you decide to go for it, that everyone tries to prescribe it [smart inhalers]. And I think that it should be clear who people should turn to in case of problems. At least, someone should be responsible.”*	HCP
**Theme 3: Feasibility**		
11	*“I wouldn’t necessarily have a problem with it [using the app] being every day, as long as it really doesn’t take much time. If you can just, say, with one push on a button register “okay, I’ve now taken my medication”, that’s okay, if that happens every day. But you don’t want to spend ten extra minutes every day. Because it is something that is part of your routine […] Then I don’t have time for that [using the app] and I don’t feel like it.”*	Patient
12	*“I’m always very concerned about the amount of time it would take if I were to prescribe it to every patient. Not to mention the costs. Because if the app doesn’t work, or the device doesn’t work, or the patient doesn’t understand, they will come to me with all those questions. […] So, I’m worried about the time investment of the doctor or the practice nurse.”*	HCP
13	*“I think, it is useful if a company start this [developing smart inhalers], that they show responsibility regarding reuse of materials, or collection and careful processing; the environmental aspect. […] I think companies could distinguish themselves positively with that, and that it is very important.”*	HCP
14	*“What I think is important: is the world ready? […] And if I look at, for example, patients with COPD as a target group. I don’t know. Somehow it seems like the target audience just isn’t ready for that [implementation of smart inhalers] yet. Or wants to use it. […] That could also have something to do with it. That the moment just hasn’t come yet.”*	Developer
15	*“How do you get these devices, these digital solutions in the hands of patients? Like, in practice, how does that work? Because a drug you just write on a piece of paper or on your digital tool. The fact that, you know, there is a certain code for the drug and then the person goes to the pharmacy and gets the drug and everything is automatic, right? I mean, there is an entire industry behind it that moves drugs around, that allows patients to get access to these drugs. It’s not the same for these solutions [smart inhalers].”*	Developer
**THEME 4: Payment and reimbursement**		
16	*“I don’t think I would use it if it was very expensive. Because I think my asthma medication is expensive enough.”*	Patient
17	*“I don’t know if it is a onetime fee […]. People might be willing to pay some money for it, but they shouldn’t have to buy a new smart inhaler every time they receive a new inhaler.”*	HCP
18	*“So, it’s like: do you want to spend a lot of money on it or not. If you don’t know whether something will work, then of course, you don’t want to spend too much on it. See, if you know it really suits you and it really helps you, then you’re willing to pay more than if it’s completely unknown.”*	Patient
19	*“Well, a lot of innovations lead to less production. And less production means less money. And then of course, the old way of thinking is that you pay for treatments, and that must change somehow. A new way of financing must be created.”*	Policy maker
**Theme 5: Data safety and ownership**		
20	*“You know, if it [smart inhaler] can only be dispensed by medical personnel, then you assume it [data privacy] is all covered and that there is no commercial interest in distributing my medical data.”*	Patient
21	*“But it is patient data. And what do they [manufacturers] do with it? How safe is that? And whose data is it anyway? In the end, they [manufacturers] use it, so I think it is an ethical issue.”*	Policy maker
22	*“For example, we noticed that the patient [who used a smart inhaler] took it [medication] every time at 4 am and 2* *pm. So, it turned out that he went out every night. It can be annoying for many people if they have to share that with someone.”*	HCP
23	*“I’m wondering to what extent it is feasible for a doctor to keep track of all data in such a portal. I think it would be nice [to share data], for example, when you have an asthma exacerbation or the medication regimen isn’t quite right. But I think the doctor receives a lot of useless information when things are going well.”*	Patient
24	*“I think it could be used well to start a conversation with the patient about its use [of medication]. What I often see with glucose monitors in diabetics, which is of course something that people keep track of themselves and that [data] is not sent to HCPs, that it does help to get insight. If they [patients] say “well, here’s the data, what do you think?” you can continue with that.”*	HCP
25	*“I think I would like to share it [data], because it is an extra push that I take it [medication]. Because for me it is very often the case that when I think: “I’m doing very well, I don’t really have any symptoms”, that I think: “Okay, I would like to halve my dose”. And then [when data is shared] we can discuss: “how is it going and is it possible [to change dose]?”.”*	Patient

*HCP* healthcare professional.

### Theme 1: Perceived benefits

The benefits that patients and HCPs expect from using a smart inhaler are dependent on the availability of certain functionalities. A list of all functionalities that emerged during the focus group discussions and interviews is presented in Table 5. Perceived benefits of smart inhaler use by patients included improved medication adherence and improved inhalation technique. HCPs reported improved medication adherence, time savings due to less consultations with stable patients and improved inhalation technique as potential benefits.

**Table 5:** Functionalities identified as a facilitator to use smart inhalers by patients, HCPs, and developers.

Participant group	Functionality
Patients	• Dose counting (i.e. knowing when it’s time to replace the inhaler) • Receiving feedback on inhalation technique • Set daily medication reminders, especially when symptom-free (i.e. easier to forget to inhale) • Receive missed-dose messages and be able to adapt the time interval • Track symptoms with a self-determined frequency
HCPs	• Weather forecasts linked to GPS (i.e. mist, pollen) • Automatic messages advising to contact HCP when patients are not inhaling well or not feeling well • Automatic messages advising to inhale more when experiencing symptoms • Integrate questionnaires that can be completed prior to a consultation (i.e. save time)
Developers	• Medication reminders supported by behavioural change modules (e.g. motivating messages, overuse messages) • Possibility to track medication use • Possibility to track triggers and symptoms

*HCP* healthcare professional.

Besides the perceived benefits mentioned, reasons were cited for not offering smart inhalers to all patients with asthma. HCPs highlighted that smart inhalers should only be prescribed if patients are motivated to use them. This was also cited by two developers, who stressed that HCPs are the ones to motivate patients, and that smart inhalers should be prescribed to patients who expect benefits from its use. One developer hypothesised that, for example, well-controlled asthma could be a reason for patients not to use a smart inhaler. This did not emerge during the patient focus group discussion, although one patient indicated that tracking symptoms would not be something she would do when her asthma is well-controlled. Higher age was suggested by a policy maker as a potential reason for patients not to use smart inhalers, which was also mentioned by one patient who reported higher age and the lack of need for change (i.e. being satisfied with current healthcare) to be reasons not to use a smart inhaler. One HCP shared the perception that patients do not want to be constantly confronted with their disease, as a barrier to smart inhaler use (Q1, Table 3). One policy maker argued that alternatives to smart inhalers are available, and that improvement of asthma care could probably be achieved if HCPs would only prescribe a limited number of inhaler devices and one type of inhaler device per patient.

Availibility of evidence was brought up as a facilitator for implementation by all participant groups, although each group defined “evidence” differently. Two patients indicated that they would only use or buy a smart inhaler if their HCP supports or works with smart inhalers, implying that support from HCPs is perceived as evidence (Q2). HCPs pointed out that proven effectiveness on outcomes such as improved asthma control or improved self-management, is important in facilitating inclusion of smart inhalers in clinical guidelines, which would then convince HCPs to prescribe smart inhalers. For policy makers, evidence meant demonstration of added value of smart inhalers for individual health, population health and cost reduction (i.e. cost-effectiveness analysis). Furthermore, data on which subgroup of patients benefit from the use of smart inhalers, the necessary duration of use (i.e. start-stop criteria) and feasibility of implementation is needed for successful implementation according to policy makers (Q3).

### Theme 2: Usability

#### User-friendliness

All participant groups cited “user-friendliness” as a facilitator for the implementation of smart inhalers (Q4). Components contributing to user-friendliness that were reported included: (i) inhaling should not be more complicated with an attached or integrated smart inhaler (e.g. more inspiratory flow needed, harder to activate the device) compared to a usual inhaler, (ii) push messages should not have a pedantic character and must be limited in frequency, (iii) installing and using the app and portal should be intuitive and should not require much effort, (iv) prescribing smart inhalers should be easy, and (v) data should be collected passively, with as little effort as possible by patients and HCPs (i.e. no technical issues, no manual data uploads). In addition, the possibility to personalise the app was cited as a facilitator by patients, HCPs and developers (e.g. possibility to change timing and frequency of reminders), as it could avoid annoyance and increase alignment with treatment goals (Q5). Developers considered it necessary to involve all stakeholders including the end-user in the development process, to improve the usability (Q6).

#### Compatibility

A barrier to implementation cited by all participants groups was the lack of compatibility on different levels. First, patients can use more than one inhaler and patients who switch inhalers should still be able to use a smart inhaler. This means that smart inhaler devices need to be compatible with different inhalers or smart inhalers apps should be compatible. One HCP commented that it is better to develop a few well-functioning smart inhalers, than one for every available inhaler (Q7, Q8). Second, smart inhaler portals should be compatible (e.g. open platform), as HCPs treat patients using different inhalers and they do not want to use a different portal for each inhaler. Third, the integration of smart inhaler data in electronic health records (EHRs) would facilitate the preparation of patient consultations. Finally, the current lack of compatibility between EHRs of healthcare institutions makes it hard for developers to create an EHR integrated smart inhaler.

#### Education and support

Patients expressed different preferences regarding support for setting up and using the smart inhaler for the first time. They ranged from electronic instructions present in the app and video instructions, to face-to-face instructions. Patients who preferred face-to-face instructions emphasised that they would prefer to receive the instructions from their treating HCP. Furthermore, patients indicated that they prefer to contact their HCP rather than a helpdesk in case of problems (Q9), because of the established patient-HCP relationship. HCPs suggested that their whole team should be trained in the use of smart inhalers and that there should be a clear protocol. Unlike patients, HCP suggested that in case of problems a helpdesk or one person in the organisation (e.g. student or researcher) should be available and responsible (Q10). Developers reported that “not supporting” is a critical barrier to adoption, which is why it is important to provide training to HCPs as they are the ones that communicate with patients. Additionally, a helpdesk and troubleshooting material for HCPs and intuitive instructions in the app were considered supportive by developers.

### Theme 3: Feasibility

A much-discussed topic was whether patients and other stakeholders believed that implementing smart inhalers is feasible. A number of facilitators and barriers with regard to feasibility emerged. Although there were differences in perceptions on acceptable time investment, patients reported that it should be minimal and it has to fit in their daily routines (Q11). A HCP reported that it seems not feasible to prescribe a smart inhaler to all patients with asthma due to limited consultation time/length, but it would be better manageable when smart inhalers are prescribed to a small target group (Q12). A HCP and a policy maker pointed out that developers should show responsibility regarding the environmental aspects of the production, processing and reuse of materials (Q13), and that a lack of environmental sustainability could influence willingness to use smart inhalers.

Agenda setting was suggested to be facilitating for implementation by policy makers, developers and HCPs. This could comprise collaboration between patient organisations and HCP groups to get the problem of non-adherence in respiratory diseases and smart inhalers on the agenda of policy makers, or increasement of visibility (e.g. by presenting on respiratory conferences for HCPs). Furthermore, a developer suggested the provision of a (national) platform for smart inhalers by policy makers to facilitate the implementation. A barrier to agenda setting that emerged was the legislation regarding contacting patients by developers to promote use of smart inhalers, which is not allowed in the Netherlands.

A policy maker indicated that far too little implementation research has been performed over the last decades, leading to insufficient consideration of implementation barriers. For example, he mentioned the importance of the willingness of healthcare organisations to change clinical workflows, as implementing innovations require other competences (such as remote coaching), and adjustment in workflows (e.g. remote monitoring instead of regular consultations). In addition, one developer wondered if now is the right time for implementation, implying the need for a feeling of readiness to implement digital technologies (Q14). A reason cited by developers for not being ready is that the development of a reliable and safe medical device evolves more slowly than expected, due to several reasons (Box 1).

Box 1 Reasons brought up by developers for delay in the implementation of smart inhalers in daily practice, ranked by frequency of occurrence

1. Time needed to develop a reliable and safe solution.
2. Time needed to collect sufficient data and to prove effectiveness.
3. The lack of knowledge and experience among developers regarding the implementation, distribution and the reimbursement of eHealth solutions (Q15).
4. Time needed to collaborate with stakeholders such as other developing companies.
5. Time needed to involve stakeholders such as HCPs and insurance companies in the development and implementation process.
6. The lack of a straightforward regulatory framework regarding implementation of medical devices that are combined with a drug.
7. The lack of a distribution and payment infrastructure for digital technologies.

### Theme 4: Payment and reimbursement

Lack of reimbursement (i.e. patients would have to pay for the device themselves) was considered to be a barrier to the implementation of smart inhalers by patients and HCPs (Q16, Q17). Some patients indicated that they were willing to pay a small one-time fee, but only if smart inhalers have been proven to be effective for them (Q18). The need for a clearly defined target group to minimise costs, was highlighted by policy makers. Furthermore, it was added that reimbursement will only be considered if effectiveness of smart inhalers (i.e. on outcomes such as asthma control, quality of life or improved self-management) is shown. Another barrier to implementation emphasised by a policy maker is the current financial incentive in the Dutch healthcare system (i.e. face-to-face consultations with patients), in which there is no room for technological innovations because they could reduce the number of consultations. As a result, implementation could be blocked by healthcare organisations when it turns out that production declines due to the innovation (Q19). Also, it is difficult to decide and prioritise which technology should be reimbursed, as technologies are evolving very quickly and it is hard to keep track for health insurers and other policy makers. One developer did not consider reimbursement a requirement for implementation, as commercial sale is also an option. Another developer stressed that it is important that HCPs will be reimbursed for invested time.

### Theme 5: Data safety and ownership

This theme addressed the ethical question who owns the data, as well as data sharing between HCPs and patients, and the privacy and security of smart inhaler data. Knowing who has access to data and the guarantee that data is properly secured was found to be important to patients. Additionally, patients highlighted that they perceive a smart inhaler to be unsafe with regard to data privacy when it is sold online, because there may be commercial interest (Q20). Devices are considered safer when prescribed by HCPs. This barrier was also brought up by policy makers, who expressed scepticism towards the development of devices for inhalers whose patents have expired (or are about to expire), as the development appears to be driven by commercial interest. Furthermore, policy makers recognised the added value of digital tools, but they considered the uncertainty about what developers will do with, and who has access to data as a barrier to implementation. It could be used to benefit the community, but concerns were expressed about potential disadvantages for patients (Q21). Developers indicated that they understand that data privacy and ownership is an important topic for HCPs and patients and that it is their job to explicitly describe what the purpose of the data is, who has access to it, and where it is stored, and to ensure that this is clear to patients.

Data sharing between patients and HCPs was considered as a barrier on the one hand and as a facilitator on the other. A reason to not share data with the HCP, is that patients consider it personal information. This was corroborated by HCPs, who mentioned that sharing data is something patients should decide for themselves (Q22). Furthermore, patients described that they doubt the feasibility of processing such a large amount of data by HCPs and wondered to which extent HCPs can keep track of the data (Q23). Suggestions that were made are sharing information only during consultations by showing the app to the HCP, or to offer the possibility to enable data sharing only when patients experience symptoms (Q24). However, patients also indicated that they do not always know whether they are taking the correct dose and whether their inhalation technique is correct, and that sharing this data could help them to discuss this with their HCP and to stay controlled or become better controlled (Q25). HCPs emphasised that data sharing could be used to start a discussion on medication use with a patient.

## Discussion

This qualitative study provides insights into the perceptions of relevant stakeholders regarding the implementation of smart inhalers in the healthcare system, and the anticipated barriers and facilitators of importance in its success. In total, we identified five themes covering 32 facilitators, including the need for evidence, the willingness to change workflows by healthcare organisations and the involvement of all stakeholders in the development process, and 14 barriers, including lack of reimbursement, lack of integration of smart inhaler data in EHRs and uncertainty regarding data access.

All participant groups brought up the need for evidence, but the meaning of the word “evidence” differed per group. For example, HCPs mentioned the need for added value on the level of clinical outcomes, whereas patients perceived support for smart inhalers by their HCP as evidence. The lack of evidence on mHealth interventions (i.e. the use of mobile and wireless technologies to benefit health outcomes) more generally has previously been described as a barrier for implementation^24,26,39^. Collecting sufficient and high-quality evidence on the effectiveness of EMDs and other mHealth interventions is complicated, as digital technologies evolve fast, whereas research to collect evidence is a time consuming process. A lack of evidence on effectiveness seems to have influence on other identified barriers, such as the lack of interest to use smart inhalers in case of insufficient evidence, and decisions regarding reimbursement of smart inhalers (i.e. no reimbursement by policy makers in the absence of evidence), and is therefore one of the key barriers found in this study^40,41^.

Several developers and policy makers brought up the importance of involvement of end-users in all development phases (i.e. user-centred or participatory design methods) to improve usability^42^. Unfortunately, only a small proportion of digital health application publications seem to report on usability outcomes^43^. Moreover, questionnaires are mostly used, which provide only an overall measure^43^. Therefore, it should be recommended to follow reporting guidelines when reporting on eHealth solutions, such as the CONSORT-EHEALTH checklist which includes usability^44^. One contributing factor to the lack of usability in the case of smart inhalers, is the lack of compatibility at different levels. Our finding of the lack of integration of smart inhaler data in electronic medical registry systems is corroborated by previous studies^23,27,39^. Additionally, stakeholders in this study reported a lack of uniformity between smart inhalers, which could result in using multiple smart inhalers and apps with different functionalities by patients who use more than one inhaler or patients who switch inhalers.

Besides involving end-users to improve usability, using participatory design methods may help tackle workflow issues. The willingness to change workflows by healthcare organisations was brought up as an important factor for the implementation of smart inhalers by a policy maker. Granja et al. identified “workflow” as the most relevant factor determining the success or failure of eHealth interventions in a systematic review. Barriers associated with “workflow” included workload, workflow disruption, alignment with clinical processes, undefined and changed roles, undermined face-to-face communication, and staff turnover^45^. To be able to address these, it could be beneficial to involve end-users in the development process.

With regard to the feasibility of implementation of smart inhalers, it was pointed out by a HCP that smart inhaler manufacturers could positively distinguish themselves by showing responsibility for the environmental aspects of production, processing, and reuse of materials. Although the environmental sustainability issue has only been addressed by a healthcare professional and a policy maker, it is an important topic, given the current global focus on climate change and the fact that smart inhalers contain electric circuits and batteries. Howard et al. also identified environmental impact as a barrier to the use of smart inhalers in their Delphi survey^26^. Recycling and rechargeable devices could be options to minimise waste and exhaustion of valuable resources. One could think of a collective effort of patients taking their used devices to pharmacies, pharmacies collecting the devices and sending them to the manufacturers, and manufacturers recycling the materials.

Reimbursement was a much-discussed topic, with each stakeholder group having its own interests. For example, policy makers require evidence on the added value of smart inhalers to enable reimbursement, HCPs want to be compensated for their time invested and patients want to pay as little as possible. A complicating factor regarding reimbursement decisions is that eHealth use by HCPs is difficult to quantify and therefore does not easily fit in the current reimbursement system in healthcare^46,47^. A second complicating factor is the perspective regarding alternatives to smart inhalers. A policy maker reported that prescribing a limited number of inhalers and only one type of inhaler per patient could be an alternative as it could potentially improve asthma outcomes, while HCPs included a previous study reported no alternatives to smart inhalers^27^. A third factor that plays a role in reimbursement decisions is the fast evolvement of technique, which complicates decision-making for policy makers.

Data privacy and data access is a topic considered important by all participant groups. Our findings resemble the findings of previous studies, in which lack of security and privacy and confidentiality issues have been described as barriers to the use of smart inhalers^25,39,46^. The concerns about data privacy and access expressed by stakeholders are not unfounded, as it was found that only 67% of all medical mHealth applications provided privacy policies, and only 55% of the all medical mHealth applications complied with their privacy policy^40^. Developers included in this study indicated that is their task to ensure that data privacy and access are explicitly described for patients and HCPs. Contradictive perceptions towards data sharing (i.e. being facilitative or hindering), have been described previously^26,41^. By offering the possibility to personalise the application, including the option to enable and disable data sharing, this barrier could be addressed.

A strength of this study is the inclusion of representatives from multiple relevant stakeholder groups including developers of smart inhalers. While developers play a major role in designing smart inhalers, and in addressing certain barriers regarding the use and implementation of smart inhalers (e.g. usability issues and environmental sustainability), they have not previously been included in qualitative studies of smart inhalers and their implementation. In addition, at least one HCP was included from all relevant professions (e.g. GP, pulmonologist, pulmonary nurse, pharmacist, paediatrician) in the HCP focus group discussion. Furthermore, our study provides in-depth information on perceived/anticipated facilitators and barriers to the implementation of smart inhalers, which is important for supporting successful implementation in the healthcare system.

The study has several limitations. First, focus group discussions with patient and HCPs were not repeated, which makes it uncertain whether data saturation is reached. However, the results show the different perspectives of a broad sample of stakeholders on the little-researched topic of smart inhalers. Second, patients and HCPs with interest in the topic of smart inhalers may have been more engaged with their asthma treatment and more inclined to participate in the study. Given less engaged patients may also have more problems with adherence, and all patients were female, one should be cautious with generalising these patient opinions. In line with this, given that patient recruitment took place via social media, this may have resulted in a sample that is more technology minded. Third, we included a patient group with, according to ACQ-5 score, generally mild to moderate symptoms, and mostly had childhood onset of asthma. These patients may have relatively easy-to-treat asthma and may need less adherence support than patient with more difficult-to-treat asthma. Further information on asthma phenotype and medical history could potentially more insights in patient’s motivation variation but was not collected. Fourth, all patient participants and the majority of the participating HCPs and policy makers had no experience in using a smart inhaler, which implies that our data is based on expectations of smart inhalers. This is mostly due to the fact that smart inhalers are developed very recently and have not yet been widely implemented. Looking at Rogers’ theory of diffusions of innovations, this means only innovators and early adopters will have experience in using smart inhalers (mainly in a research context)^48^. Fifth, note that the participants were all Dutch and extrapolating findings across countries should be done with caution. However, a recent UK user experience study regarding digital inhalers found several similar issues (such as data security) raised by patients^49^. Finally, we planned to conduct all interviews and focus group discussions face-to-face, but this was not feasible due to the Covid-19 pandemic. Therefore, focus group discussions and semi-structured interviews were conducted online using video conferencing. The benefits of remote focus groups discussions and interviews we experienced were flexibility for participants (i.e., no travel time) and the possibility to recruit without a geographic barrier, whereas disadvantages were limitations in the interpretation of non-verbal communication, and less discussion than expected (i.e. consensus was reached relatively quickly for some topics). Other possible factors influencing remote focus group discussions that have been described previously are the increased perceived level of privacy, the lack of technical skills as a barrier to participate and a limited personal connection^50^.

The barriers and facilitators identified in this study are relevant to policy makers and developers involved in the implementation of smart inhalers. It is important to involve stakeholders, including patients and HCPs, in the design and development of smart inhalers so that the barriers presented can be addressed and implementation challenges overcome. Key factors that should be addressed prior to large-scale implementation include the lack of evidence of effectiveness and target groups, usability issues, workflow changes in healthcare organisations due to implementation, compatibility issues on different levels, the lack of reimbursement agreements and clarity regarding data access and security. The results of this study could contribute to the design of an implementation strategy and may help developers in improving the design of the current devices, which together could increase the chance of successful implementation.

## Supplementary information

Supplementary Material

Reporting Summary

## Data Availability

The data that support the findings of this study are available from the corresponding author upon request. All data provided will be anonymised and terms for usage will be agreed in advance.
