# Supplementary material for: Anticipated barriers and facilitators for implementing smart inhalers in asthma medication adherence management
Source: NPJ Prim Care Respir Med. 2023 May 20;33:22. doi: 10.1038/s41533-023-00343-w (PMC10198788; doi:10.1038/s41533-023-00343-w)
Supplement: Supplementary file 1 — Supplementary Material [file 41533_2023_343_MOESM1_ESM.pdf]

SUPPLEMENTARY INFORMATION

1. Focus group guides

Interview guide for patient focus group (translated from Dutch to English)

Introduction

1. Introduction question: experience
   a. What do you think of digital tools in healthcare in general (such as: pedometer, online consultation scheduling, video consultation)? Why do you think that?
   b. Do you have experience with the use of a digital tool?
      i. What kind of experience?
      ii. What was this experience like?

2. Introduction question: smart inhalers
   a. Did you ever hear or read about smart inhalers?
      i. What did you hear about it?
      ii. Where did you hear/read about smart inhalers?

3. Needs
   a. Would you have a need for a smart inhaler? Why (not)?
   b. I have mentioned some optional functionalities of smart inhalers in the introduction (such as insight in when and how often you use and should use your inhaler (according to the prescription), monitoring inhaler technique, monitoring of asthma symptoms, set reminders). Which functionalities would you like, or do you find useful?
      i. Why do you find those functionalities useful?
   c. Would you share information about your inhaler use with your doctor during a consultation? Why (not)?
   d. What do you think of the doctor being able to access information about your inhaler use remotely via an online portal? Why?
   e. What do you think should be the purpose of the smart inhaler? Why do you think that?

4. Motivation for use
   When smart inhalers become available in the healthcare system and the doctor can prescribe them:
   a. What would be your reasons to use a smart inhaler? Which considerations play a role?
   b. What would be your reasons to not use a smart inhaler? Which considerations play a role?
   c. How much time would you spend on using a smart inhaler?

10-minute break

5. Concerns
   a. When smart inhalers become available in the healthcare system and the doctor can prescribe them, are there things that worry you? Why?

6. Support
   a. When smart inhalers become available in the healthcare system and the doctor can prescribe them, would you like help or education?
      i. What kind of information do you want?
      ii. In what way?
   b. From whom would you like to receive information? Why?
   c. Who would you contact if the smart inhaler is not working properly? Why?

7. Costs
    a. If smart inhalers can be ordered (online) without a doctor's prescription, would you buy it? Why (not)?
    b. How much do you think you would pay for it? Why?

Final remarks and thank participants for participation

<u>Interview guide for healthcare professionals focus group (translated from Dutch to English)</u>

Introduction

1. Introduction question:
    a. What do you know about smart inhalers?
        i. How did you hear about smart inhalers?
        ii. Did any of your patients ever ask about smart inhalers?

2. Motivation for use
    a. What would be reasons to start using a smart inhaler in the treatment of asthma? What are your considerations?
    b. What would be reasons to not use a smart inhaler in the treatment of asthma? What are your considerations?

3. Barriers
    a. Besides smart inhalers, do you think other methods or developments exist that could be used in asthma treatment to achieve the same goal as smart inhalers?
        i. If yes: which methods/developments?
        ii. Do you expect a smart inhaler to be more effective or less effective? Why?
    b. What do you think are the challenges for the implementation of the smart inhaler in the Dutch healthcare system?
        i. Can these challenges be overcome? How?
        ii. Which functionalities do you consider important and essential for the adoption?

10-minute break

4. Facilitating factors
    a. What do you think are factors that promote the implementation of the smart inhaler in the Dutch healthcare system?

5. Implementation
    a. Who do you think will ensure that smart inhalers can be used in practice?
    b. What steps do you think need to be taken to implement smart inhalers in the healthcare system?
        i. What should be arranged in your own practice or hospital to actually use the smart inhaler in asthma care?
    c. What do you think is the main purpose of smart inhalers?
    d. Do you think it is feasible to implement smart inhalers on a large scale?
        i. What is required for that? Why?

Final remarks and thank participants for participation

2. Semi-structured interview guides

Interview guide for policy makers (translated from Dutch to English)

1. Introduction question
   - Could you briefly introduce yourself? Who are you, what is your profession and where do you work?
     - Did you ever hear about smart inhalers?
     - What did you hear about it?
     - What is your connection to smart inhalers?

2. Facilitating factors
   a. How could smart inhalers be implemented in the Dutch healthcare system?
      ii. Is it possible to implement smart inhalers in Dutch healthcare via different routes?
      iii. Which routes?
      iv. Which route do you prefer?
   b. Who do you think will ensure that smart inhalers can be implemented in the Dutch healthcare system?
      i. Which institutes and ministries play a role?
      ii. What is their role?
      iii. What does their role entail?
   c. What steps do you think need to be taken to implement smart inhalers in the healthcare system?
      i. Why?
      ii. In what order?
      iii. What steps does your organisation/ministry/institute have an influence on?
      iv. How?
      v. For which steps is your organization/ministry/institute dependent on others? From whom?
      vi. What steps could healthcare professionals and developers take?
   d. What information is needed to be able to carry out the steps mentioned before?
      i. Why?
      ii. Who will review or assess this information?
      iii. How will this information be assessed?
      iv. Does the assessment for add-on devices differ from the assessment of integrated devices?
      v. How is it determined whether a medical device is covered by basic or supplementary insurance?
      vi. Is expected adoption part of the assessment?
   e. Who will eventually buy smart inhalers (e.g., pharmacies, hospitals, patients, government)
      i. Who will determine the price of smart inhalers?
      ii. What is this price based on?

3. Barriers
   a. What do you think could be reasons for the smart inhaler not yet being implemented?
      i. Why do you think that?
      ii. Can these issues be overcome?
      iii. How or why not?
      iv. Does legislation play a role?

4. Adoption
   a. Many innovative medical devices are being developed. How do you determine the value difference?

5. Future
    a. If smart inhalers are implemented in the healthcare system, will implementation be evaluated?
        i. Why (not)?
        ii. By who?
        iii. What will be evaluated?
        iv. Who is responsible for adjustments?
        v. Does the outcome of the evaluation determine whether the medical device can remain in the health care system?
        vi. How will this be determined?
    b. What do you think is needed to implement smart inhalers on a large scale in the Netherlands? Why?
    c. The implementation of smart inhalers will change the workflow for healthcare providers. How much time do you think it would take before the smart inhaler is widely used in the treatment of asthma?
        i. On what does this depend?

Interview guide for developers (translated from Dutch to English)

1. Introduction question
    a. Could you briefly introduce yourself? Who are you, what is your profession and where do you work?
    b. How are you involved with smart inhalers?
    c. How do smart inhalers fit within your company?

2. Goal
    a. What is, from your perspective, the main purpose of smart inhalers?
        i. Why do you think this is the main purpose?
        ii. What is the target group? Why?
        iii. Is the target group involved in the development and implementation process? Why (not)?
        iv. Are other stakeholders involved in the development and implementation process?
        v. What is the role of healthcare providers?
        vi. Besides smart inhalers, do you think other methods or developments exist that could be used in asthma treatment to achieve the same goal as smart inhalers?
            - If yes: which methods/developments? Do you expect a smart inhaler to be more effective or less effective? Why?
    b. Which functionalities do you think are essential?
        i. Why do you think these functionalities are important?
        ii. Are these functionalities already available? If not: why not?
        iii. Do you think these functionalities are already optimized?
        iv. Is your company unable to integrate some functionalities (that other smart inhalers do have) because of IP rights?

Barriers
    a. What do you think are the main reasons for the smart inhaler not yet being implemented in the Dutch healthcare system?
    b. What do you think are the main barriers for the implementation of the smart inhaler?
        i. Why are those barriers?
        ii. Do you think these barriers can be overcome?
            - If yes: how
            - If no: why not?
        iii. Does legislation play a role?

Facilitating factors
    a. Who do you think will ensure that smart inhalers can be used in practice?

   i. Which and how many organisations play a role?
   ii. What role?
b. How could smart inhalers be implemented in the Dutch healthcare system?
   i. Is it possible to implement smart inhalers in Dutch healthcare via different routes?
   ii. Which routes?
   iii. Which route do you prefer?
c. Who will eventually buy smart inhalers (e.g., pharmacies, hospitals, patients, government)
   i. Who will determine the price of smart inhalers?
   ii. What is this price based on?
d. What steps do you think need to be taken to implement smart inhalers in the healthcare system?
   vii. Why?
   viii. In what order?
   ix. What steps does your company have an influence on?
   x. How?
   xi. For which steps is your company dependent on others? From whom?

Adoption

a. The implementation of smart inhalers will change the workflow for healthcare providers. How much time do you think it would take before the smart inhaler is widely used in the treatment of asthma?
   i. On what does this depend?
   ii. What is this expectation based on?
   iii. Is support offered to promote implementation and adoption? Why (not)?
   iv. To whom is support offered?
   v. In what manner?

Future

a. What do you think is needed to implement smart inhalers in the Netherlands?
   i. Why?
   ii. What do you think is needed to implement smart inhalers on a large scale in the Netherlands?

3. Final coding tree

1. **Overall evaluation of (digital) gadgets**
    - 1.1. Data sharing
    - 1.2. Evaluation of digital tools/devices
    - 1.1. Familiar with smart inhalers
    - 1.2. Need for smart inhalers

2. **Barriers**
    - 2.1. Costs
    - 2.2. HCPs
        - 2.2.1. Data sharing
        - 2.2.2. Lack of compatibility
        - 2.2.3. Lack of motivation
        - 2.2.4. Lack of time/time investment
        - 2.2.5. Privacy of data
    - 2.3. Manufacturers
        - 2.3.1. Collaboration with other stakeholders
        - 2.3.2. Development time
        - 2.3.3. Lack of compatibility between (smart) inhalers
        - 2.3.4. Lack of evidence
        - 2.3.5. Lack of knowledge/experience
        - 2.3.6. Legislation
    - 2.4. Patient organizations
        - 2.4.1. Commercial interest
        - 2.4.2. Reimbursement system
    - 2.5. Patients
        - 2.5.1. Costs
        - 2.5.2. Data sharing
        - 2.5.3. Lack of motivation
        - 2.5.4. Lack of usability
            - 2.5.4.1. Lack of compatibility between (smart) inhalers
        - 2.5.5. Privacy policy
            - 2.5.5.1. Commercial interest
        - 2.5.6. Time investment
    - 2.6. Regulators/insurers
        - 2.6.1. Alternative options
        - 2.6.2. Commercial interest
        - 2.6.3. Lack of compatibility
            - 2.6.3.1. Between EPDs
        - 2.6.4. Lack of knowledge/evidence
        - 2.6.5. Privacy policy
        - 2.6.6. Reimbursement system
    - 2.7. Timing / readiness

3. **Facilitators**
    - 3.1. Agenda setting
    - 3.2. HCPs
        - 3.2.1. Compatibility between smart inhaler (portals)
        - 3.2.2. Data sharing
        - 3.2.3. Education/support
        - 3.2.4. Evidence
        - 3.2.5. Guidelines
        - 3.2.6. Integration with EPD
        - 3.2.7. Motivation
        - 3.2.8. Usability
    - 3.3. Manufacturers
        - 3.3.1. Collaboration with stakeholders
        - 3.3.2. Evidence/knowledge on experiences
        - 3.3.3. Involvement of end-user in development
        - 3.3.4. Quantity/device specifications
        - 3.3.5. Reimbursement
        - 3.3.6. Sustainability
    - 3.4. Patients
        - 3.4.1. Compatibility between (smart) inhalers
        - 3.4.2. Data sharing
        - 3.4.3. Education/support
        - 3.4.4. Evidence/used by HCPs
        - 3.4.5. Motivation/need
        - 3.4.6. Personalised
        - 3.4.7. Privacy policy
        - 3.4.8. Usability
    - 3.5. Regulators/insurers
        - 3.5.1. Evidence
            - 3.5.1.1. Clinical outcomes
            - 3.5.1.2. Cost-effectiveness
            - 3.5.1.3. Target group
        - 3.5.2. Guidelines
    - 3.6. Smart inhaler functionalities
        - 3.6.1. Dose counter
        - 3.6.2. Feedback on inhalation technique
        - 3.6.3. HCP portal
        - 3.6.4. Monitor disease control and triggers
        - 3.6.5. Monitor medication adherence
        - 3.6.6. Personalised
        - 3.6.7. Real-time feedback/advice
        - 3.6.8. Reminders
        - 3.6.9. Stimuli and allergies

4. **Goal**
    - 4.1. Functionalities
        - 4.1.1. Data sharing
    - 4.2. Incentive
        - 4.2.1. Financial
        - 4.2.2. Inhalation technique
        - 4.2.3. Time saving
        - 4.2.4. Insight in (patient) behaviour
        - 4.2.5. Medication adherence
        - 4.2.6. Outcomes
        - 4.2.7. Patient empowerment
    - 4.3. Target group
        - 4.3.1. Involvement of end-user in development

5. **Implementation steps**
    - 5.1. Distribution
    - 5.2. Payment
    - 5.3. Regulatory frameworks
    - 5.4. Roles of stakeholders
    - 5.5. Scale-up
