# Supplementary material for: Anticipated barriers and facilitators for implementing smart inhalers in asthma medication adherence management
Source: NPJ Prim Care Respir Med. 2023 May 20;33:22. doi: 10.1038/s41533-023-00343-w (PMC10198788; doi:10.1038/s41533-023-00343-w)
Supplement: Supplementary file 2 — Reporting Summary [file 41533_2023_343_MOESM2_ESM.pdf]

nature portfolio

Corresponding author(s): J.F.M. van Boven

Last updated by author(s): May 2, 2023

Reporting Summary

Nature Portfolio wishes to improve the reproducibility of the work that we publish. This form provides structure for consistency and transparency in reporting. For further information on Nature Portfolio policies, see our Editorial Policies and the Editorial Policy Checklist.

Statistics

For all statistical analyses, confirm that the following items are present in the figure legend, table legend, main text, or Methods section.

n/a	Confirmed	
☒	☐	The exact sample size (*n*) for each experimental group/condition, given as a discrete number and unit of measurement
☒	☐	A statement on whether measurements were taken from distinct samples or whether the same sample was measured repeatedly
☒	☐	The statistical test(s) used AND whether they are one- or two-sided<br>*Only common tests should be described solely by name; describe more complex techniques in the Methods section.*
☒	☐	A description of all covariates tested
☒	☐	A description of any assumptions or corrections, such as tests of normality and adjustment for multiple comparisons
☒	☐	A full description of the statistical parameters including central tendency (e.g. means) or other basic estimates (e.g. regression coefficient) AND variation (e.g. standard deviation) or associated estimates of uncertainty (e.g. confidence intervals)
☒	☐	For null hypothesis testing, the test statistic (e.g. *F*, *t*, *r*) with confidence intervals, effect sizes, degrees of freedom and *P* value noted<br>*Give P values as exact values whenever suitable.*
☒	☐	For Bayesian analysis, information on the choice of priors and Markov chain Monte Carlo settings
☒	☐	For hierarchical and complex designs, identification of the appropriate level for tests and full reporting of outcomes
☒	☐	Estimates of effect sizes (e.g. Cohen's *d*, Pearson's *r*), indicating how they were calculated

Our web collection on statistics for biologists contains articles on many of the points above.

Software and code

Policy information about availability of computer code

Data collection	No software was used.
Data analysis	All transcripts were coded using Dedoose software (version 9.0).

For manuscripts utilizing custom algorithms or software that are central to the research but not yet described in published literature, software must be made available to editors and reviewers. We strongly encourage code deposition in a community repository (e.g. GitHub). See the Nature Portfolio guidelines for submitting code & software for further information.

Data

Policy information about availability of data

All manuscripts must include a data availability statement. This statement should provide the following information, where applicable:

- Accession codes, unique identifiers, or web links for publicly available datasets
- A description of any restrictions on data availability
- For clinical datasets or third party data, please ensure that the statement adheres to our policy

Provide your data availability statement here.

Research involving human participants, their data, or biological material

Policy information about studies with human participants or human data. See also policy information about sex, gender (identity/presentation), and sexual orientation and race, ethnicity and racism.

Reporting on sex and gender	Only females participants with asthma participated in the focus group; sex was not considered in the study design (i.e., everybody with self-reported asthma could participate).
Reporting on race, ethnicity, or other socially relevant groupings	We did not use any socially constructed categorization variables in our manuscript.
Population characteristics	See below.
Recruitment	Patients with asthma were recruited through social media advertisement. Upon expression of interest, a patient information sheet was sent by email. The sheet contained information about the concept 'smart inhaler', the purpose of the study and inclusion criteria, it explained that participation is voluntary and can be ended at any time, that the focus group discussion would be audio-recorded and that data will be used confidentially and anonymously. As recruitment took place via social media, this may have resulted in a sample that is more technology minded.
Ethics oversight	The medical ethics committee of the University Medical Center Groningen (UMCG) deemed that formal medical ethical approval was not required, as this study did not fall under the Dutch Medical Research Involving Human Subjects Act (METc number 2020/145).

Note that full information on the approval of the study protocol must also be provided in the manuscript.

Field-specific reporting

Please select the one below that is the best fit for your research. If you are not sure, read the appropriate sections before making your selection.

☐ Life sciences ☒ Behavioural & social sciences ☐ Ecological, evolutionary & environmental sciences

For a reference copy of the document with all sections, see nature.com/documents/nr-reporting-summary-flat.pdf

Behavioural & social sciences study design

All studies must disclose on these points even when the disclosure is negative.

Study description	Qualitative study
Research sample	Four groups of participants, representing different key stakeholders involved in the implementation of smart inhalers, were included: (i) patients with asthma, (ii) Healthcare professionals, (iii) policy makers (i.e. representatives from public health institutions/decision makers, health insurance companies and patient organisations), and (iv) smart inhaler developers (i.e. representatives from pharmaceutical companies and medical device companies involved in the development and manufacturing of smart inhalers). Only female patients with asthma participated.
Sampling strategy	Selection of policy maker and developer representatives was based on their function within the company or institution (i.e. most knowledgable on the topic of interest). Purposive sampling was used to select HCPs for the focus group discussion (e.g. occupation). Convenient sampling was used to select patients with asthma (i.e. all eligible patients with asthma that were interested could participate). Using an extensive single focus group session for patients and for HCPs, it was anticipated that a sufficient level of data saturation would be reached. However, data saturation may have been slightly increased by repeating the focus groups with other patients and HCPs.
Data collection	The focus group discussions were held using Zoom and led by NS (moderator, 'science business and policy' master student) with SvdH (general practitioner in training and PhD-student) and BFdB (PhD) assisting in data collection (observers). The moderator and observers had no involvement in patient care of the participating patients. The participants of both focus group discussions had no personal background information on the interviewers (other than a brief introduction during the focus group discussions), except for one HCP who was a known contact of one of the observers. Field notes were made during the focus group discussions. Focus group discussions were recorded (via Zoom).<br>The interviews were conducted by NS using Zoom as well, except for one telephonic interview with a participant that worked in a company that did not support video calling.
Timing	Data was collected in the period from April 2020 to June 2020.
Data exclusions	No data were exlcuded from the analysis.
Non-participation	In total, 20 patients with asthma were interested in participation, 13 met inclusion criteria and nine participated in the focus group

Non-participation	discussion (four did not reply to our follow-up e-mails). Invitations were sent to 20 HCPs to which 17 responded and seven were able to participate in the focus group discussion.
Randomization	Participants were not allocated to experimental groups.

Reporting for specific materials, systems and methods

We require information from authors about some types of materials, experimental systems and methods used in many studies. Here, indicate whether each material, system or method listed is relevant to your study. If you are not sure if a list item applies to your research, read the appropriate section before selecting a response.

Materials & experimental systems

n/a	Involved in the study
☒	☐ Antibodies
☒	☐ Eukaryotic cell lines
☒	☐ Palaeontology and archaeology
☒	☐ Animals and other organisms
☒	☐ Clinical data
☒	☐ Dual use research of concern
☒	☐ Plants

Methods

n/a	Involved in the study
☒	☐ ChIP-seq
☒	☐ Flow cytometry
☒	☐ MRI-based neuroimaging
